# Low Tunneling Decay of Iodine-Terminated Alkane Single-Molecule Junctions

**DOI:** 10.1186/s11671-018-2528-z

**Published:** 2018-04-24

**Authors:** Lin-Lu Peng, Bing Huang, Qi Zou, Ze-Wen Hong, Ju-Fang Zheng, Yong Shao, Zhen-Jiang Niu, Xiao-Shun Zhou, Hu-Jun Xie, Wenbo Chen

**Affiliations:** 1 Key Laboratory of the Ministry of Education for Advanced Catalysis Materials, Institute of Physical Chemistry, Zhejiang Normal University, Jinhua, 321004 Zhejiang China; 2 Shanghai Key Laboratory of Materials Protection and Advanced Materials in Electric Power, Shanghai University of Electric Power, Shanghai, 200090 China; 3 Department of Applied Chemistry, Zhejiang Gongshang University, Hangzhou, 310018 China

**Keywords:** Electron transport, Barrier height, Single molecular junction, Iodine, Alkyl-based molecules

## Abstract

One key issue for the development of molecular electronic devices is to understand the electron transport of single-molecule junctions. In this work, we explore the electron transport of iodine-terminated alkane single molecular junctions using the scanning tunneling microscope-based break junction approach. The result shows that the conductance decreases exponentially with the increase of molecular length with a decay constant *β*_N_ = 0.5 per –CH_2_ (or 4 nm^−1^). Importantly, the tunneling decay of those molecular junctions is much lower than that of alkane molecules with thiol, amine, and carboxylic acid as the anchoring groups and even comparable to that of the conjugated oligophenyl molecules. The low tunneling decay is attributed to the small barrier height between iodine-terminated alkane molecule and Au, which is well supported by DFT calculations. The work suggests that the tunneling decay can be effectively tuned by the anchoring group, which may guide the manufacturing of molecular wires.

**Electronic supplementary material:**

The online version of this article (10.1186/s11671-018-2528-z) contains supplementary material, which is available to authorized users.

## Background

Understanding the electron transport of single-molecule junctions is crucial for the development of molecular electronic devices [1–16]. The non-resonant tunneling model has often been used to describe the electron transport process through small molecule, where contact conductance, molecular length, and the tunneling decay constant are the main parameters [17, 18]. In most molecular systems, decay constant is highly related to the electronic properties of organic backbone. For example, the conjugated molecular systems have low tunneling decay, unlike non-conjugated ones [17, 19]. Since the tunneling decay is decided by the barrier height between the Fermi level of electrode and lowest unoccupied molecular orbital (LUMO) or highest occupied molecular orbital (HOMO) of molecular junctions [17, 20], it is possible to tune the molecular energy level towards the Fermi level to achieve the low decay [21–24].

In single-molecule junctions, the anchoring group plays an important role in the control of electronic coupling between the organic backbones with the electrodes [21, 23–25]. A series of conductance measurements for the alkane-based molecules have showed a significant effect of different anchoring groups on the binding geometry, junction formation probabilities, contact conductance, and even conductance channel (through LUMO or HOMO) of molecular junctions [21–25]. Since the anchoring group can regulate the frontier orbitals in the molecular junction, the tunneling decay of the molecule may also be tuned by the anchoring group [24]. However, limited study has been focused on this area.

**Fig. 1:**
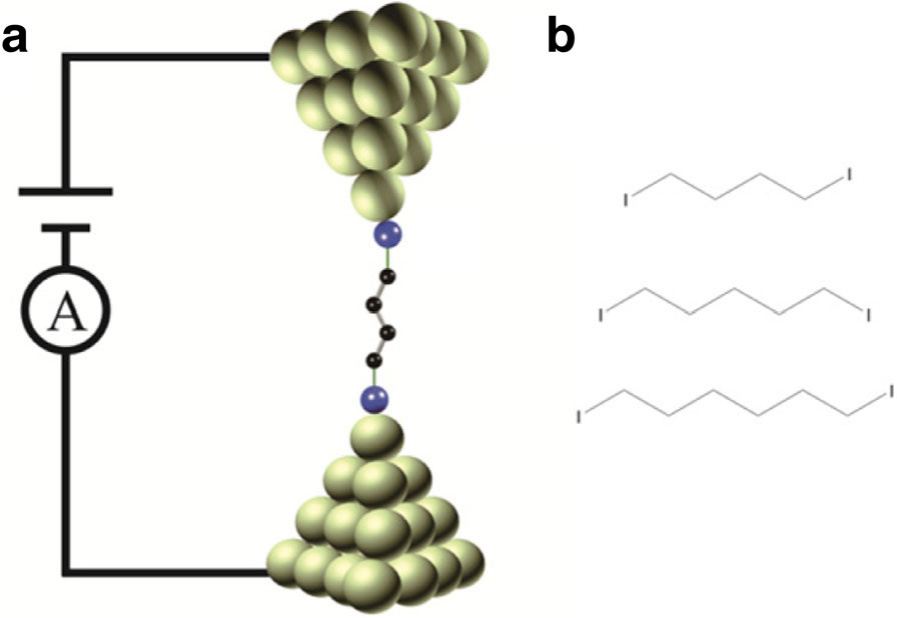
Schematic diagram of scanning tunneling microscopy break junction (STM-BJ) and molecular structures. **a** Schematic of the STM-BJ with molecular junction. **b** Molecular structures of alkane iodine molecules

Herein, we report the electron transport of alkane molecules terminated with iodine group by using scanning tunneling microscopy break junction (STM-BJ) (Fig. 1) [26, 27]. The single molecular conductance measurements show that the conductance decreases exponentially with the increase of molecular lengths and the decay constant of alkane molecules with iodine group is much lower than that of the analogues with other anchoring groups. The different tunneling decay constants for alkane molecules with varied anchoring groups are explained by barrier height between molecule and electrode.

## Methods

1,4-Butanediiodo, 1,5-pentanediiodo, and 1,6-hexanediiodo were purchased from Alfa Aesar. All solutions were prepared with ethanol. Au(111) was used as the substrate, while mechanically cut Au tips were used as the tips. Before each experiment, the Au(111) was electrochemically polished and carefully annealed in a butane flame and then dried with nitrogen.

The Au(111) substrate was immersed into a freshly prepared ethanol solution containing 0.1 mM target molecules for 10 min. The conductance measurement was carried out on the modified Nanoscope IIIa STM (Veeco, USA.) by using the STM-BJ method at room temperature [28–30], which simply measured the conductance of single-molecule junctions formed by repeatedly moving the tip into and out of the substrate at a constant speed. During the process, the molecules could anchor between the two metal electrodes and form single molecular junctions. Thousands of such curves were collected for statistical analysis. All the experiments were performed with a fix bias voltage of 100 mV. Since molecules with iodine as the anchoring group are a photosensitive material, the experiment was carried out under shading.

## Results and Discussion

### Conductance Measurement of Iodine-Terminated Alkane Single Molecular Junctions

**Fig. 2:**
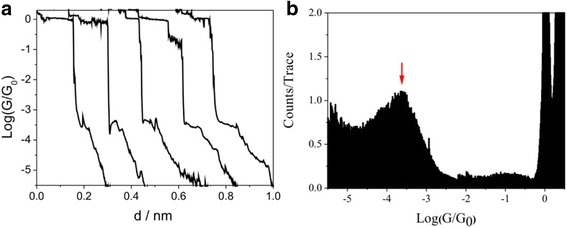
Single molecular conductance of Au–1,4-butanediiodo–Au junctions. **a** Typical conductance curves of Au–1,4-butanediiodo–Au junctions measured at a bias of 100 mV. **b** Log-scale conductance histogram of 1,4-butanediiodo junctions with Au contacts

The conductance measurements were first carried out on Au(111) with monolayer of 1,4-butanediiodo by STM-BJ. Figure 2a gives out the typical conductance traces exhibiting the stepwise feature. Conductance traces show plateau at 1 *G*_0_, indicating the formation of stable Au atomic contact. Plateau at a conductance value of 10^−3.6^ *G*_0_ (19.47 ns) is also found besides the 1 *G*_0_, owing to the formation of molecular junction. A conductance histogram could also be obtained by treating with logarithm and binning of conductance value from more than 3000 conductance traces, and then, the intensity of conductance histogram was normalized by the number of traces used and shows a conductance peak at 10^−3.6^ *G*_0_ (19.44 ns) (Fig. 2b). Those show that the iodine group can serve as an effective anchoring group forming molecular junction. However, this value is smaller than the single molecular conductance value of 1,4-butanediamine with amine as the anchoring group, which may stem from weak interaction between iodine and Au electrode [31].

**Fig. 3:**
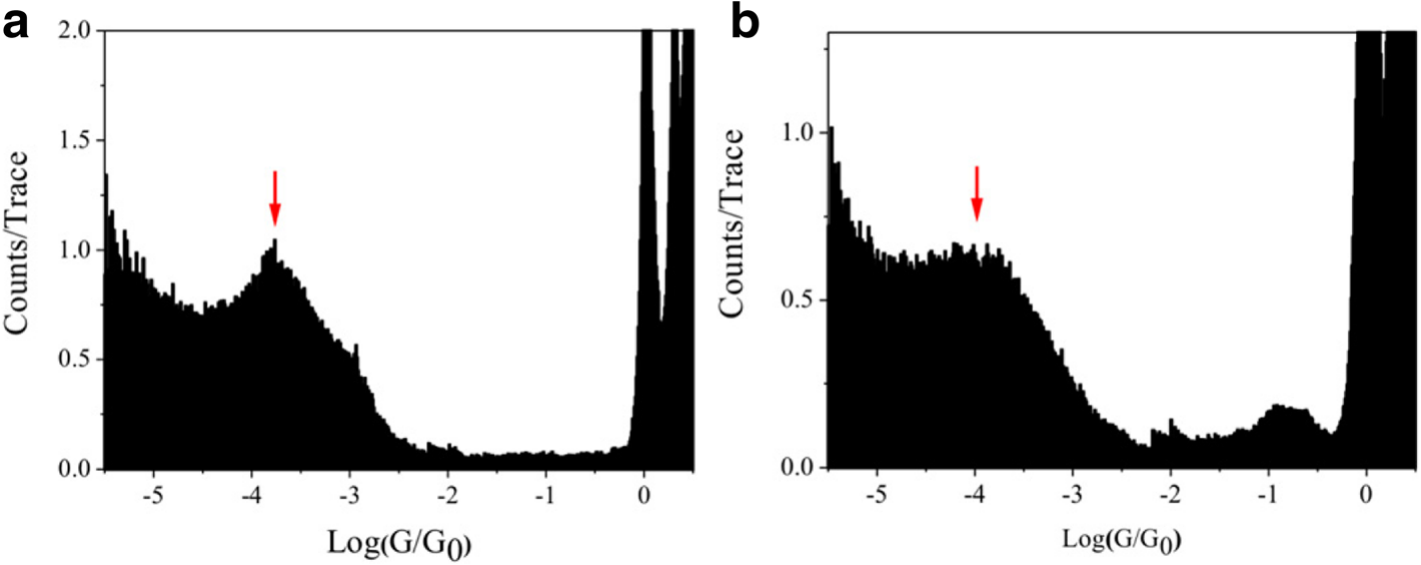
Single molecular conductance of 1,5-pentanediiodo and 1,6-hexanediiodo with Au electrode. Log-scale conductance histogram of single molecular junctions with **a** 1,5-pentanediiodo and **b** 1,6-hexanediiodo

In comparison with 1,4-diiodobutane, pronounced peaks at 10^−3.8^ *G*_0_ (12.28 ns) and 10^−4.0^ *G*_0_ (7.75 ns) are found for 1,5-pentanediiodo and 1,6-hexanediiodo, respectively (Fig. 3). The conductance values decrease with the increasing of molecule length. Meanwhile, the conductance values of 1,5-pentanediiodo and 1,6-hexanediiodo are smaller than those of 1,5-pentanediamine and 1,6-hexanediamine, respectively [31], which may be caused by the different interaction in alkane-based molecular junctions between iodine and amine anchoring groups binding to Au electrodes [32].

**Fig. 4:**
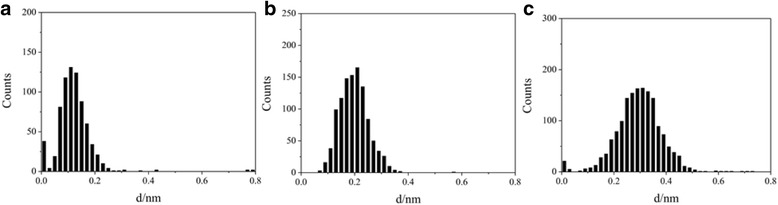
Breaking off distances for iodine-terminated alkanes. Breaking off distances of **a** 1,4-butanediiodo, **b** 1,5-pentanediiodo, and **c** 1,6-hexanediiodo obtained from conductance curves between 10^−5.0^ *G*_0_ and 10^−0.3^ *G*_0_

The two-dimensional conductance histograms were also constructed for those molecular junctions (Additional file 1: Figure S1) and give out similar conductance values of one-dimensional histograms. Typically, the breaking off distance of molecular junctions increases with the increasing of molecular length. We also analyze the distance from the conductance value of 10^−5.0^ *G*_0_ to 10^−0.3^ *G*_0_ as shown in Fig. 4, and rupture distances of 0.1, 0.2, and 0.3 nm are found for 1,4-butanediiodo, 1,5-pentanediiodo, and 1,6-hexanediiodo, respectively. Here, the rupture distances are obtained from the maximum peak of the rupture distance histogram [33]. It was reported that there is a snap back distance of 0.5 nm for Au after the breaking of Au–Au contact [34, 35]; thus, the absolute distances for those molecular junctions between electrodes could be 0.6, 0.7, and 0.8 nm which are found for 1,4-butanediiodo, 1,5-pentanediiodo, and 1,6-hexanediiodo, respectively. Those distances are comparable to the length of molecules. Eder et al. reported that the adsorption of 1,3,5-tri (4-iodophenyl)-benzene monolayer onto Au(111) may cause partial dehalogenation [36]; however, a very larger conductance value for those Au–C covalent contact molecular junctions can be found for molecules with four (around 10^−1^ G_0_) and six (bigger than 10^−2^ G_0_) –CH_2_– units [37]. Thus, we propose that the current investigated molecules contact to the Au through the Au–I contact.

### Tunneling Decay Constant of Iodine-Terminated Alkane Single Molecular Junctions

**Fig. 5:**
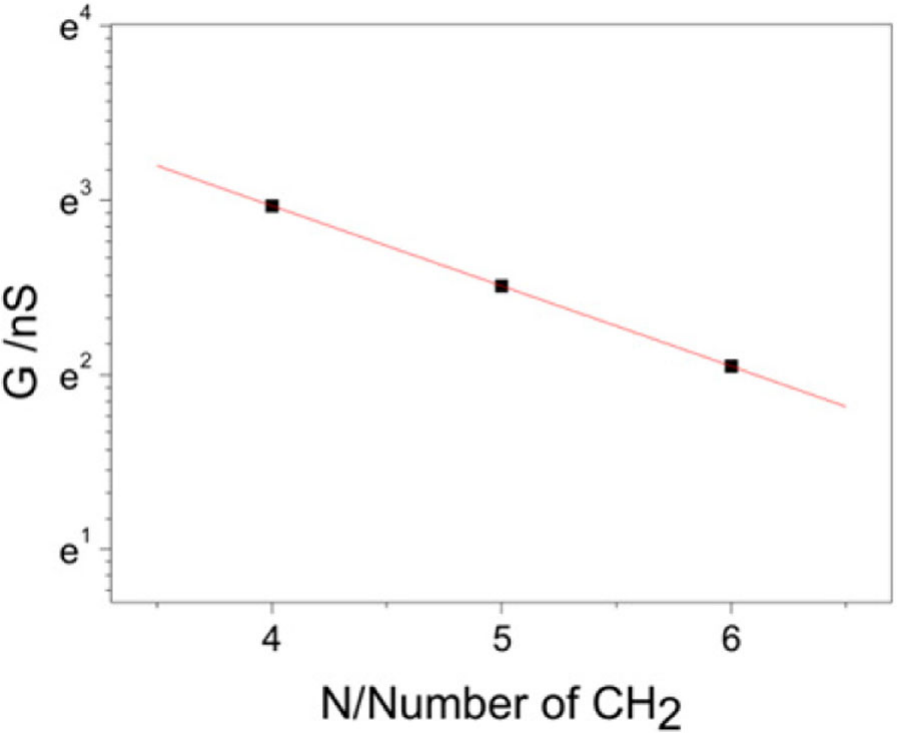
Single-molecule conductance vs molecular length for iodine-terminated alkanes. Logarithmic plots of single-molecule conductance vs molecular length for iodine-terminated alkanes

Under the current bias, those molecule conductance can be expressed as *G* = *G*c exp(–*β*_N_*N*). Here, *G* is the conductance of the molecule and *G*c is the contact conductance and is determined by the interaction between the anchoring group and the electrode. *N* is the methylene number in the molecule, and *β*_N_ is the tunneling decay constant, which reflects the coupling efficiency of electron transport between the molecule and the electrode. As show in Fig. 5, we plot a natural logarithm scale of conductance against the number of methylene; tunneling decay constant *β*_N_ of 0.5 per –CH_2_ is determined from the slope of linear fitting. This tunneling decay is very low in alkane-based molecules. For the alkane-based molecules, *β*_N_ is usually found around 1.0 per –CH_2_ for thiol (SH) [23, 38], while around 0.9 and 0.8 per –CH_2_ are determined for amine (NH_2_) [23, 31] and carboxylic acid (COOH), respectively [39]. Thus, the tunneling decay with iodine shows the lowest value among those anchoring groups with a trend *β*_N_ (thiol) > *β*_N_ (amine) > *β*_N_ (carboxylic acid) > *β*_N_ (iodine), which may be due to the difference in the alignment of molecular energy levels to the Fermi level of Au electrode [23, 31]. The tunneling decay of 0.5 per –CH_2_ can also be converted to 4 nm^−1^, which is comparable to oligophenyls with 3.5–5 nm^−1^ [40, 41].

The *β*_N_ for the metal-molecule-metal junctions can be simply described by the below equation [17, 20, 38],

$$ {\beta}_N\ \alpha\ \sqrt[2]{\frac{2 m\varPhi}{h^2}} $$

where *m* is the effective electron mass and  is the reduced Planck’s constant. *Φ* represents the barrier height, which is decided by the energy gap between the Fermi level and the molecular energy levels in the junction. Obviously, the *β*_N_ value is proportional to the square root of barrier height. Thus, we may propose that iodine-terminated alkane molecules have small *Φ* with the Au electrode.

### Barrier Height of Single Molecular Junctions with Different Anchoring Groups

Taking the –(CH_2_)_6_– as the backbone, we performed the rough calculations (see computational detail in Additional file 1) to investigate the frontier molecular orbitals of complexes with four Au atoms at the both ends, including 1,6-hexanedithiol (C6DT), 1,6-hexanediamineb (C6DA), 1,6-hexanedicarboxylic acid (C6DC), and 1,6-hexanediiodo (C6DI). As shown in Table 1, the HOMO and LUMO are − 6.18 and − 1.99 eV, respectively, for C6DT, while HOMO (6.02 eV) and LUMO (− 1.85 eV) are found for C6DA. Meanwhile, HOMO and LUMO energy levels are calculated for C6DC (-6.33 and -2.58 eV) and C6DI (-6.22 and -2.61 eV).

**Table 1:** Energy levels of the frontier orbitals of molecules contacting with four Au atoms computed by DFT method

	Au_4_-C6DT-Au_4_ (eV)	Au_4_-C6DA-Au_4_ (eV)	Au_4_-C6DC-Au_4_ (eV)	Au_4_-C6DI-Au_4_ (eV)
E_LUMO_	− 1.99	− 1.85	− 2.58	− 2.61
E_HOMO_	− 6.18	− 6.02	− 6.33	− 6.22
E_LUMO_-E_Au_	2.21	2.35	1.62	1.59
E_Au_-E_HOMO_	1.98	1.82	2.13	2.02

For the Fermi level of Au electrode, we need to consider the influence of the adsorption of molecules. In the vacuum condition, clean Au gives out work function of 5.1 eV [42]; meanwhile, this value can be obviously changed by the adsorption of molecules. Kim et al. [43] and Yuan et al. [44] have found that the work function of Au is around 4.2 eV (4.0–4.4 eV) upon the adsorbed self-assembled monolayers (SAMs) measured by the ultraviolet photoelectron spectrometer (UPS). Low et al. also investigated the electron transport of thiophene-based molecules of TOTOT (LUMO − 3.3 eV, HOMO − 5.2 eV) and TTO_p_TT (LUMO − 3.6 eV, HOMO − 5.1 eV) with Au as the electrode (T, O, and O_p_ denote thiophene, thiophene-1,1-dioxide, and oxidized thienopyrrolodione, respectively) [45]. The results show that the Fermi level of Au is in the middle of LUMO and HOMO. Thus, we can infer the Fermi level of Au can be around the average energy level of LUMO and HOMO, which are − 4.25 and − 4.35 eV established from TOTOT and TTO_P_TT, respectively. The Fermi level of Au − 4.25 and − 4.35 eV are similar to that measured by UPS with − 4.2 eV [43]. According to the above, we will use the − 4.2 eV as the Fermi level of Au electrode with the adsorption of molecule.

Assuming the Fermi level of − 4.2 eV for Au with SAM, C6DT and C6DA are the HOMO-dominated electron transport, while LUMO-dominated electron transport is proposed for the C6DC and C6DI. Thus, the barrier height *Φ* can be established as 1.98 eV (C6DT), 1.82 eV (C6DA), 1.62 eV (C6DC), and 1.59 eV (C6DI) (Table 1). The trend for the barrier height between the molecule and Au is *Φ*_C6DT_ (thiol) > *Φ*_C6DA_ (amine) > *Φ*_C6DC_ (carboxylic acid) > *Φ*_C6DI_ (iodine), which is consistent with the trend of the tunneling decay (*β*). Thus, the unusual low tunneling decay can be contributed to the small barrier height between iodine-terminated alkane molecules and Au.

## Conclusions

In conclusion, we have measured the conductance of alkane-based molecules with iodine group contacting to Au electrodes by STM-BJ at room temperature. A tunneling decay *β*_N_ of 0.5 per –CH_2_ was found for those molecules with Au electrodes, which is much lower than that of alkane-based molecules with other anchoring groups. This can be caused by the small barrier height between the iodine-terminated alkane molecule and Au. The current work shows the important role of the anchoring group in electrical characteristics of single molecular junctions, which can tune the tunneling decay of molecular junction and guide the manufacturing molecular wire.

## Additional file

Additional file 1: Two-dimensional conductance histograms of molecular junctions and computational details. (DOCX 173 kb)

## Abbreviations

HOMO: Highest occupied molecular orbital
LUMO: Lowest unoccupied molecular orbital
SAMs: Self-assembled monolayers
STM-BJ: Scanning tunneling microscopy break junction
UPS: Ultraviolet photoelectron spectroscopy
