# Supplementary material for: Low Tunneling Decay of Iodine-Terminated Alkane Single-Molecule Junctions
Source: Nanoscale Res Lett. 2018 Apr 24;13:121. doi: 10.1186/s11671-018-2528-z (PMC5972139; doi:10.1186/s11671-018-2528-z)
Supplement: Supplementary file 1 — Two-dimensional conductance histograms of molecular junctions and computational details. (DOCX 173 kb) [file 11671_2018_2528_MOESM1_ESM.docx]

**Low Tunneling Decay of Iodine-terminated Alkane Single-Molecule Junctions†**

Lin-Lu Peng^1^, Bing Huang^1^, Qi Zou^2^, Ze-Wen Hong^1^, Ju-Fang Zheng^1^, Yong Shao^1^, Zhen-Jiang Niu^1^, Xiao-Shun Zhou^1*^, Hu-Jun Xie^3*^, Wenbo Chen^2*^

^1^Key Laboratory of the Ministry of Education for Advanced Catalysis Materials, Institute of Physical Chemistry, Zhejiang Normal University, Jinhua, Zhejiang, 321004, China.

E-mail: xszhou@zjnu.edu.cn (X.-S.Z)

^2^Shanghai Key Laboratory of Materials Protection and Advanced Materials in Electric Power, Shanghai University of Electric Power, Shanghai 200090, China.

E-mail: wenbochen@shiep.edu.cn (W.C)

^3^Department of Applied Chemistry, Zhejiang Gongshang University, Hangzhou 310018, China. E-mail: hujunxie@gmail.com (H.-J.X)


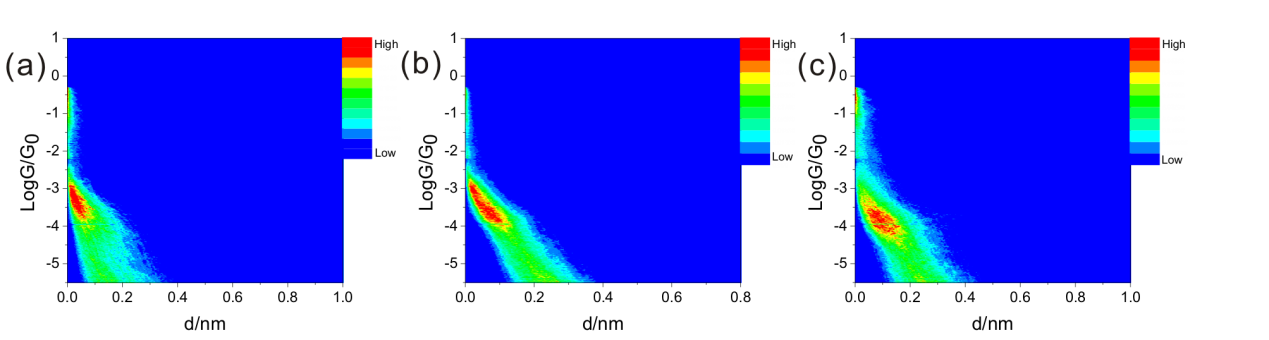


**Figure S1.** Two-dimensional conductance histograms of 1,4-butanediiodo, 1,5-pentanediiodo and 1,6-hexanediiodo contacting to Au electrode.

**DFT calculation detail**

Taking the -(CH_2_)_6_- as the backbone, we performed the rough DFT calculations to investigate the frontier molecular orbitals of complexes with four Au atoms at the both ends, including 1,6-hexanedithiol (C6DT), 1,6-hexanediamineb(C6DA), 1,6-hexanedicarboxylic acid (C6DC) and 1,6-hexanediiodo (C6DI). All species in this study were fully optimized by means of the DFT calculations at the M06-2X level.[^1^](#_ENREF_1)^,^ [^2^](#_ENREF_2) The 6-31G(d,p) basis set[^3^](#_ENREF_3)^,^ [^4^](#_ENREF_4) was used for the C, H, O and N atoms, while the effective core potentials (ECPs) of Hay and Wadt with a double-ζ valence basis set (LanL2DZ)[^5^](#_ENREF_5)^,^ [^6^](#_ENREF_6) were selected to describe the Au, S, and I atoms. Furthermore, the polarization functions of Au(ζ_f_) = 1.050, I(ζ_d_) = 0.266, and S(ζ_d_) = 0.421 were added.[^7^](#_ENREF_7)^,^ [^8^](#_ENREF_8) Frequency calculations have also been performed to validate the stationary points as minima. All calculations were implemented via the Gaussian09 software package[^9^](#_ENREF_9).

**References**

1. Y. Zhao and D. G. Truhlar, *J. Phys. Chem. A*, 2006, **110**, 5121-5129.

2. Y. Zhao and D. G. Truhlar, *J. Phys. Chem. A*, 2006, **110**, 13126-13130.

3. M. S. Gordon, *Chem. Phys. Lett.*, 1980, **76**, 163-168.

4. R. C. Binning and L. A. Curtiss, *J. Comput. Chem.*, 1990, **11**, 1206-1216.

5. P. J. Hay and W. R. Wadt, *J. Chem. Phys.*, 1985, **82**, 270-283.

6. P. J. Hay and W. R. Wadt, *J. Chem. Phys.*, 1985, **82**, 299-310.

7. S. Huzinaga, J. Andzelm, M. Klobukowski, E. Radzioandzelm, Y. Sakai and H. Tatewaki, *Gaussian basis sets for molecular calculations*, Elsevier, Amsterdam, 1984.

8. A. W. Ehlers, M. Böhme, S. Dapprich, A. Gobbi, A. Höllwarth, V. Jonas, K. F. Köhler, R. Stegmann, A. Veldkamp and G. Frenking, *Chem. Phys. Lett.*, 1993, **208**, 111-114.

9. M. J. Frisch, G. W. Trucks, H. B. Schlegel, G. E. Scuseria, M. A. Robb, J. R. Cheeseman, G. Scalmani, V. Barone, B. Mennucci, G. A. Petersson, H. Nakatsuji, M. Caricato, X. Li, H. P. Hratchian, A. F. Izmaylov, J. Bloino, G. Zheng, J. L. Sonnenberg, M. Hada, M. Ehara, K. Toyota, R. Fukuda, J. Hasegawa, M. Ishida, T. Nakajima, Y. Honda, O. Kitao, H. Nakai, T. Vreven, J. A. Montgomery Jr., J. E. Peralta, F. Ogliaro, M. J. Bearpark, J. Heyd, E. N. Brothers, K. N. Kudin, V. N. Staroverov, R. Kobayashi, J. Normand, K. Raghavachari, A. P. Rendell, J. C. Burant, S. S. Iyengar, J. Tomasi, M. Cossi, N. Rega, N. J. Millam, M. Klene, J. E. Knox, J. B. Cross, V. Bakken, C. Adamo, J. Jaramillo, R. Gomperts, R. E. Stratmann, O. Yazyev, A. J. Austin, R. Cammi, C. Pomelli, J. W. Ochterski, R. L. Martin, K. Morokuma, V. G. Zakrzewski, G. A. Voth, P. Salvador, J. J. Dannenberg, S. Dapprich, A. D. Daniels, Ö. Farkas, J. B. Foresman, J. V. Ortiz, J. Cioslowski and D. J. Fox, *Journal*, 2009.
